# Legal reflections on the case of genome-edited babies

**DOI:** 10.1186/s41256-020-00153-4

**Published:** 2020-05-14

**Authors:** Shuang Liu

**Affiliations:** European Law Research Center, Henan University, No.85, Minglun Road, Sunhe District, Kaifeng, 475001 China

**Keywords:** Genome-edited babies, Crime, Law, Regulation, Guideline, Global registry

## Abstract

Human genome-editing is banned by guidelines, laws and regulations in most countries. However, the first criminal case on genome-edited babies was sentenced in China in 2019. In this commentary we discuss our legal reflections on this case. Genome-editing on healthy embryos of human may lead to irreversible mutations and serious consequences on the heredity of future generations, while its long-term safety is unpredictable. A full set of laws, regulations along with the guidelines should be formulated to penalize genome-editing behaviors and prevent similar negative events in the future. More effective and binding mechanisms should be constructed and implemented among different countries. A collaborative network should be strengthened for better global registry and surveillance of human genome-editing technologies and research.

## Introduction

On December 30, 2019, a Chinese researcher, Jiankui He, was sentenced by Chinese local Court in Shenzhen City to 3 years of imprisonment with a fine of 3 million RMB Yuan for committing the crime of “Illegal Medical Practice”, and the other two defendants in the same case were also sentenced. One was sentenced to imprisonment of 2 years with a fine of 1 million RMB Yuan, another was sentenced to imprisonment of 1 year and 6 months (with probation of 2 years) with a fine of 0.5 million RMB Yuan [1]. The court concluded that each of the three defendants did not have a doctor’s practice license, and they applied the genome-editing technology (known as Clustered Regularly Interspaced Short Palindromic Repeats, CRISPR) to human assisted reproductive medicine, which caused the genetic changes of babies. In this case, the fathers are HIV positive and the mothers are HIV negative. Eggs were extracted from their body and the twin pregnancy gestated after the genome-edited embryos were transferred to their uterus. Genome-editing was undertaken to remove the CCR5 gene which allows the HIV to infect cells. The birth of babies represented a controversial leap in genome editing [2]. One day later when He announced the research of his team, more than 100 Chinese scientists and scholars signed a joint statement to denounce the trial. They reasserted that both the accuracy of the CRISPR and the potential off-target effects are controversial in the scientific community. Any attempt to directly transform human embryos and produce babies before rigorous tests poses tremendous risks. Such experiment is forbidden by the international biomedical community.

Genome-editing technology can bring great positive influence to the human being. It can be used to treat certain genome-related diseases [3]. However, it can also lead to some problems, such as how to (1) use it ethically and legally; (2) acquire the real consent from the experimental subjects; (3) how to penalize the genome-editing research for non-medical reasons, etc. Specific laws and regulations are important to resolve these problems because they are the last line of defense for social governance.

## Current laws and regulations of genome-editing in different countries

Human genome-editing is largely forbidden by laws or guidelines even in countries permissive to human embryonic stem cell research [4]. Many countries have banned human genome-editing. Thirty nine countries were surveyed and categorized as “Ban based on legislation” (25 countries), “Ban based on guidelines” (4), “Ambiguous” (9) and “Restrictive” (1). China, India, Ireland, and Japan forbid genome-editing based on guidelines which are less enforceable than laws and are subject to amendment [3]. In the USA, Human genome-editing is not banned, but a moratorium is imposed under vigilance of the Food and Drug Administration (FDA) and the guidelines of the National Institutes of Health (NIH). Any clinical trial proposals for germline alterations will be rejected by the Recombinant DNA Advisory Committee (RAC) of the NIH. Clinical studies are regulated by FDA [5]. In the UK, the legislation of medical use of mitochondrial replacement is likely to lead to legal permission for the modification of germline nuclear genome that can be readily changed by genome-editing technology [6].

Although genome-editing is banned in many countries, necessary and practical laws, regulations and guidelines should be developed, and appropriate penalty should be applied in proportion to the crime. Preventive measures should also be stipulated in a specific law. Early embryo genome-editing for fertility purposes violates the ethical principles provided in the “Declaration of Helsinki-Ethical Principles for Medical Research Involving Human Subjects” (hereafter referred to as “Declaration of Helsinki”), which has been widely accepted by the international community. In He’s case, early human embryos were edited artificially. Consequently, the genome-editing babies not only face the risk of uncertainty, but also are deprived of the right to an open future. The Article 9 of “Declaration of Helsinki” states that the responsibility for the protection of research subjects must always rest with the physicians or other health care professionals and never with the research subjects, even though they have been given consent. He and his team violated the provisions of both Article 9 of Declaration of Helsinki and the Chinese criminal law, and their misconducts should be punished.

## The sentence of genome-editing babies in China

Current Chinese laws are insufficient to deal with new challenges posed by new expertise and technologies. The regulations prohibit the development of genome-editing embryos beyond 14 days. The Chinese Guideline on Human Assisted Reproductive Technologies stipulates that the use of human egg plasma and nuclear transfer technology for the purpose of reproduction, and manipulation of the genomes in human gametes, zygotes or embryos for the purpose of reproduction are prohibited. In He’s case, it is unknown whether his team had acquired the true informed consent and they were convicted the crime of “Illegal Medical Practice”. The local court concluded that their behaviors deliberately violated the National Regulations on Scientific Research and Medical Management, crossed an ethical bottom line, and rashly applied genome-editing technology. Genome-editing on embryos with existing technologies and methods is not the only way to prevent mother-to-child transmission of AIDS [7]. Besides, the experimental procedure was unclear and nontransparent, but the consequence is full of risks. However, according to the Chinese Criminal Law, three-year imprisonment and below is regarded as misdemeanor. In contrast, the punishment of similar behavior could be 10 years of imprisonment in the UK and 20 years in France at maximum [8]. It is obvious that He and his team were eager for quick success, and their misconducts were irresponsible and dangerous. Moreover, their genome-editing behavior may cause irreversible damage to the entire human genome chain. Therefore, He’s case is very typical to warn other scientists not to commit similar misconducts. Fortunately, on May 28, 2019, the Chinese government promulgated the Regulation of the People’s Republic of China on the Administration of Human Genetic Resources, which aims to protect public health, national security, and public interest through effective protection and rational use of China’s human genetic resources.

## Conclusion

Human genome-editing technology is a two-sided sword. The advantage of its benefit can be explored. However, further legislation is required to punish misconducts and avoid potential risks.

1. A specific crime and more severe penalty should be formulated in the Chinese Criminal Law. Civil responsibility should be assumed if a medical institution or a person in charge do not truthfully and fully informed patients of potential risks.
2. Better governance is needed. According to the Administrative Penalty Law, local government and other administrative agencies should assume responsibilities if they fail to carry out their duties in ethical review, supervision and management.
3. More effective and binding mechanisms to constrain the use of genome-editing technology should be developed. More specific guidelines and preventive measures should be formulated in consistent with the international regulations.
4. A collaborative network should be strengthened for better global registry and surveillance of human genome-editing technologies and research, led by the World Health Organization (WHO) Expert Advisory Committee on Developing Global Standards for Governance and Oversight of Human Genome Editing.

## Data Availability

Not applicable.

## Abbreviations

CRISPR: Clustered Regularly Interspaced Short Palindromic Repeats.
FDA: Food and Drug Administration
NIH: National Institutes of Health
RAC: Recombinant DNA Advisory Committee
WHO: World Health Organization
